# An electrochemical nitric oxide generator for in-home inhalation therapy in pulmonary artery hypertension

**DOI:** 10.1186/s12916-022-02686-6

**Published:** 2022-12-15

**Authors:** Yiwei Liu, Yifan Zhu, Chenyu Jiang, Zhanhao Su, Yi Yan, Bei Feng, Wen Mao, Yuyan Zhang, Xiaojian Wang, Zhuoming Xu, Hao Zhang

**Affiliations:** 1 Heart Center and Shanghai Institute of Pediatric Congenital Heart Disease, Shanghai Children’s Medical Center, School of Medicine, Shanghai Jiao Tong University, Shanghai, 200127 China; 2 Shanghai Clinical Research Center for Rare Pediatric Diseases, Shanghai Children’s Medical Center, School of Medicine, Shanghai Jiao Tong University, Shanghai, 200127 China; 3 State Key Laboratory of Cardiovascular Disease, Fuwai Hospital, National Center for Cardiovascular Diseases, Chinese Academy of Medical Sciences and Peking Union Medical College, Beijing, 100037 China; 4 Institute for Cardiovascular Prevention (IPEK), Ludwig Maximilian University Munich, 80539 Munich, Germany; 5 DZHK (German Centre for Cardiovascular Research), Partner site Munich Heart Alliance, 80539 Munich, Germany; 6 Nanjing Novlead Biotechnology Corporation Limited, Nanjing, 211800 China

**Keywords:** Nitric oxide generator, Inhalation therapy, Pulmonary artery hypertension

## Abstract

**Background:**

Inhaled NO is a selective pulmonary vasodilator proven to be therapeutic for patients with pulmonary artery hypertension (PAH). The most common NO delivery system in clinical practice is cylinder-based, but unfortunately limited by its high costs, complicated delivery, and the requirement of an extensive supply chain, leaving vast unmet medical needs globally.

**Methods:**

To address the need for rapid, affordable, and safe production of nitric oxide (NO) for in-home inhalation therapy in patients with PAH. We developed a novel portable device to derive NO from a nitrite complex solution with a copper(II)-ligand catalyst, and further examined its effectiveness in a porcine model of PAH. This model was established by using female Bama miniature pig and induced by monocrotaline (MCT) administration.

**Results:**

This generator could rapidly and safely produce therapeutic NO at concentrations ranging from 0 to 100 parts per million (ppm) with the least disproportionated nitrogen dioxide (NO_2_) and byproducts. It could effectively alleviate pulmonary arterial pressure (PAP) and pulmonary vascular resistance (PVR) in piglets with PAH, without causing major physiologic disruptions.

**Conclusions:**

Our electrochemical NO generator is able to produce the desired NO doses for pulmonary vasodilation in a safe and sustainable way, with low costs, which paves the way for its subsequent clinical trials in the patient with PAH and other common cardiopulmonary conditions with a high disease burden around the world.

**Supplementary Information:**

The online version contains supplementary material available at 10.1186/s12916-022-02686-6.

## Background

Pulmonary artery hypertension (PAH) is a severe clinical syndrome characterized by progressive pulmonary vascular remodeling and increased pulmonary arterial pressure (PAP) [1]. Despite the development of novel medical therapies, the long-term prognosis of patients with PAH remains far from satisfactory, especially infants with idiopathic PAH and adults with obstinate PAH [2, 3]. Poor control of PAP is a major risk factor for early death among PAH patients. For various reasons, many patients with PAH have suboptimal control of PAP. For these PAH patients, there is an urgent need for reliable and sustainable therapies for the in-home management of pulmonary pressure.

The current first-line medications for PAH are vasoactive agents such as sildenafil, nitroprusside, and bosentan [1], but the use of these drugs is associated with systemic vasodilation that can lead to long-term arterial hypotension. Since the early 1990s, gaseous nitric oxide (NO) emerged as the first verified selective vasodilator that targets the pulmonary vasculature to exert a potent vasodilating effect. NO is generally released by endothelial cells and increases cyclic guanosine monophosphate (cGMP) levels by activating the protein kinase G (PKG) pathway, thereby relaxing pulmonary vascular smooth muscle cells without causing systemic hypotension [4, 5]. In 1999, inhaled NO was approved by the U.S. Food and Drug Administration (FDA) for the treatment of persistent pulmonary hypertension in neonates (PPHN) [6]. Recent studies have indicated that inhaled NO could also be applied in patients with ischemia–reperfusion injury [7, 8], chronic obstructive pulmonary disease (COPD) [9], insertion of a left ventricular assist device [10], intravascular hemolysis [11], and congenital heart diseases [12], as well as in COVID-19 respiratory therapy [13]. At present, cylinder-based NO delivery systems are widely used by the medical community due to their reliability and safety [14]. However, the use of NO gas cylinders has been restricted to a few inpatient clinical settings because of their bulkiness and dependence on a specialized workforce and a complicated supply network, which leads to very high medical costs [15]. Although a kind of 0.16-L miniature NO cylinder, INOpulse, has been tested in outpatient clinical scenario [16], it is still far from being widely used in in-home therapy due to its limited NO storage capacity. Indeed, treating PPHN patients with cylinder-based NO for five days can cost an average of $14,000 [14]. Therefore, NO cylinders are not suitable for conditions that require long-term management of PAH and are far less accessible in resource-limited countries.

In recent years, NO generators have emerged as an alternative to NO cylinders, such as generators based on pulse discharge or reduction of NO_2_ [17]. However, NO generators that rely on pulse discharge have not moved beyond the preclinical stage due to critical defects, such as insufficient NO production and inevitable toxic byproducts, including NO_2_/O_3_/metal brass particles. Moreover, the demand for high-voltage discharge raises safety concerns that prevent their in-home use. Although chemical-based generators are designed to deliver the desired NO concentration via the reduction of NO_2_ produced by dinitrogen tetroxide (N_2_O_4_) [18], these apparatuses are still not suitable for home therapy due to the toxicity of N_2_O_4_. Hence, electrochemical generation of NO from a compound solution has been emerging as a promising design with higher NO production capacity, lower production of toxic byproducts and lower costs.

In this study, we proposed a novel design for a portable, affordable and practical NO generator (Electrochemical NO Generator, ENG) based on electrolysis of a nitrite/Cu(II)-complex solution to generate gaseous NO, and its safety and efficacy were investigated using porcine models of PAH. This model is established by using Bama miniature pig, because its physiological features of pulmonary artery pressure and vascular resistance reduction patterns are similar to that in humans.

## Methods

### Study design

The aim of this study was to design a portable and highly integrated NO generator capable of rapidly, stably, and safely producing NO via electrochemical reduction of a low-cost nitrite/Cu(II)-ligand solution. We further connected the machine with the ventilator to monitor the real-time concentration of NO and NO_2_ in the pipeline and validate its efficacy for inhalational therapy for PAH in a porcine model. For the monocrotaline (MCT)-induced PAH model, piglets were randomized to the MCT or vehicle groups. In the study of NO treatment, animals were allocated to different groups randomly. The sample size in animal studies was determined on the basis of previous study reports and our past experience using this animal model. All in vivo experiments were conducted three times. Blinding approaches were used in histopathological analyses. The experimenters were not blinded to the identity of the study groups. The animal study conforms to the principles outlined in the ARRIVE guidelines [19].

### A dual-work mode operating system

In the first mode, nitrogen-generating membrane materials in the nitrogen (N_2_) production unit contained any one or at least two combinations of poly (4-methyl-1-pentene), brominated polycarbonate, polypropylene, polyimide, and polydimethylsiloxane. The average pore size of the nitrogen film was 0.005–0.02 μm. The flow of nitrogen was controlled by a mass flow controller at 0.2–0.5 L/min. To the solution containing 0.5 M HEPES buffer (pH 7.3) and 1 M NaNO_2_, 7 mM copper sulfate, and 7 mM Me3TACN were added as raw materials for the electrochemical generation of NO and assembled into an external electrolytic module. A 5 by 10 cm Au mesh was used as the working electrode, and it was completely immersed in the solution. The purged and filtered NO was stored in a tank with gaseous N_2_ to prevent the transformation of NO into nitrogen oxides. NO was released by mass flow controller into the NO_2_ scrubber, which comprises a carrier coated with ascorbic acid. The second mode was set by the switch valve, in which the nitrite/Cu(II)-complex solution, together with the remaining NO, was continuously circulated through a gas extraction silicone fiber dialyzer using a micro liquid pump. The residual aqueous NO was separated through the silicone fiber wall due to its high permeability and swept away by the carrier gas into the NOx absorbent.

### NO/NO_2_/O_2_ sensor

The concentration of NO/NO_2_/O_2_ was monitored by a chemiluminescence gas sensor (Honeywell, USA) integrated into the NO generator and displayed directly on the operation panel. According to the real-time monitoring data, the NO concentration can be adjusted to the therapeutic level at any time by operating the knob.

### Test of volatile organic compounds and particular matters.

According to international standard organization (ISO) 18562-3:2017, we used gas chromatograph to measure volatile organic compounds (VOCs) content at 25°C and humidity 40–60% [20]. Airflow samples were taken 100 min at the initial run, 24 h after working, and 7 days after working. A total of 10 l of gas were collected within 100 min for each VOC test sample to achieve the detection limit of 2 μg/m^3^. Other VOCs are quantified according to the ratio of peak area to toluene area. The ENG is classified as a medical device for long-term exposure use. Based on the ISO-recommended threshold of toxicological concern, we calculated the cumulative inhaled VOCs using ENG in daily breathing volumes for adults (20 m^3^/day), children (5 m^3^/day), infants (2 m^3^/day), and newborns (0.21 m^3^/day) [21]. According to ISO 18562-2:2017, laser airborne particle counter was used to measure particular matters at standard condition [22]. We set the sampling time for 24 h, recorded the particle numbers of 0.3μm, 0.5μm, 1.0μm, 2.5μm, 5.0μm and 10μm, and then converted them into particle weights per unit volume (μg/m^3^) (V=4/3*πR^3^, c=PVw/L *10^−6^, R=radius, V= volume of a single particle size, p=maximum density of sample material, w=particles number, L=gas volume, c= particle weights per unit volume).

### Animal preparation

Prior to performing experiment, we numbered the animals in column A and then grouped the animals via method of random number generated by computer (Microsoft Excel). Secondly, we used RAND() function to generated 16 different random numbers (between 0 and 1) in column B. Then we used RANK(B1, B:B) function to generate 16 different random numbers (between 1 and 16) in column C. Finally, we assigned every continuous 6 numbers of column C from top to bottom orderly into the 3 certain study groups. A total of eighteen 3-month-old healthy female Bama miniature pigs (from Wujiang Tianyu Biotechnology Co., LTD) (mean weight, 7.3±0.4 kg) were randomly divided into the control group (*n*=6), PAH group (*n*=6), and PAH+NO group (*n*=6). The animals were raised in a stainless cage separately and given qualified feed and drinking water. The temperature and humidity of the animal house were monitored, and the light and dark cycles were alternated for 12 h. Piglets in the PAH/PAH+NO group were injected intraperitoneally with MCT (12 mg/kg, dissolved in 75% alcohol) twice at an interval of 1 week, and the control group received 75% alcohol. The animal model was ready for the experiment 6 weeks after the first dose (preoperative mean weight, 14.2±1.2 kg). For MCT-induced PAH model, piglet was excluded if mean PAP < 20mmHg. Throughout the experiment, piglets were excluded if they died during the operation.

Preoperative preparations included intramuscular administration of atropine (0.02–0.04 mg/kg) to inhibit glandular secretion, Zoletil 50 (5 mg/kg) to induce anesthesia, enrofloxacin (5 mg/kg) to prevent infection, and meloxicam (0.2 mg/kg) for analgesia. Anesthesia was maintained with 1.5–3% inhaled isoflurane in 50% O_2_ through a mask connected with a ventilator and ENG. The PAH+NO group received initial inhalation of NO (20 ppm) for 4 h, and the concentration was then decreased by 5 ppm every 30 min until it finally reached 1 ppm and was maintained for 30 min. Hemodynamics were measured in anesthetized animals using a 5F Swan-Ganz catheter, and the signals were acquired by a multichannel physiological signal acquisition and processing system (RM6240E/EC, Chengdu Instrument Factory, China) at 0 h, 0.5 h, 2 h, 6 h, and 12 h after the operation. Cardiac output indicators were assessed by thermodilution, which is the mean of three measurements following rapid intravenous administration of 10 mL cold saline. Pulmonary vascular resistance (PVR), pulmonary vascular resistance index (PVRI), cardiac output (CO), and cardiac output index (CI) were calculated with standard formulas. In addition, arterial blood gas (ABL90, RADIOMETER, Denmark), routine blood parameters, biochemical parameters, coagulation factors, and methemoglobin concentrations were determined at the corresponding time points. The animals were euthanized on the scheduled autopsy day (12 h post-operation) via intravenous injection of pentobarbital sodium at a dose of 100mg/kg, followed by anatomic and histopathological examination. The right ventricular wall and lung tissues were harvested at the end of the experiment. For each experimental group, all the data points are included in the analysis.

### Histological analysis

The harvested tissues were fixed overnight in 4% paraformaldehyde. Myocardial and lung tissues were paraffin-embedded and sectioned at 5 μm. Then, the sections were stained with H&E, Verhoeff Van Gieson (EVG), and Masson using commercially available kits (Leagene) according to the manufacturer’s instructions. The histological changes were observed with a light microscope (Leica).

### Immunofluorescence staining

Immunofluorescence was performed following a standard protocol. Briefly, lung tissues were fixed in 4% paraformaldehyde. After gradient alcohol hydration, citrate (pH=6.5) was used for antigen repair of lung sections. Thereafter, the sections were blocked with 5% goat serum for 2 h and then incubated with primary antibody (rabbit anti-Ki67, 1:200, No. 27309-1-AP, Proteintech; rabbit anti-α-SMA, 1:200, No. ab5694, Abcam) at 4 °C overnight. The sections were then stained with fluorochrome-conjugated secondary antibodies for 60 min at room temperature. Images were captured with a laser-scanning confocal microscope (SP8; Leica).

### Western blot analysis

Total protein was extracted using RIPA buffer with protease inhibitors (Roche). For immunoblot analysis, protein samples were resolved by SDS–PAGE and transferred to nitrocellulose membranes with an iBlot 2 dry blotting system (Thermo Fisher Scientific). Membranes were blocked in 5% nonfat dry milk in TBST (100 mmol/L Tris, pH 7.5, 0.9% NaCl, 0.1% Tween-20) for 1 hour and incubated with primary antibodies overnight at 4 °C (rabbit anti-eNOS, 1:1000, No. ab5589, Abcam; rabbit anti-beta actin, 1:1000, No 20536-1-AP, Proteintech; rabbit anti-PKG, 1:500, No. bs-6705R, Bioss; rabbit anti-sGC, 1:500, No. bs-13544R, Bioss). The membranes were washed with TBST and incubated for 1 h with goat anti-rabbit-IgG secondary antibody (1:8000, ZSGB-Bio) at room temperature. The blots were developed using ECL (Thermo Fisher Scientific), and images were captured with a luminescent image analyzer (GE).

### Statistical analysis

All data are presented as the mean ± SD. The normality of distributions was tested with the Shapiro–Wilk test. Student’s *t* test was used to compare the means of two independent groups, and two-way ANOVA with repeated measures was used to determine the effect of breathing electrochemically generated NO on PAP and PVR (GraphPad Prism 7.0; GraphPad Software Inc.). *P* < 0.05 was considered statistically significant.

## Results

### Design and construction of a novel electrochemical NO generator

**Fig. 1:**
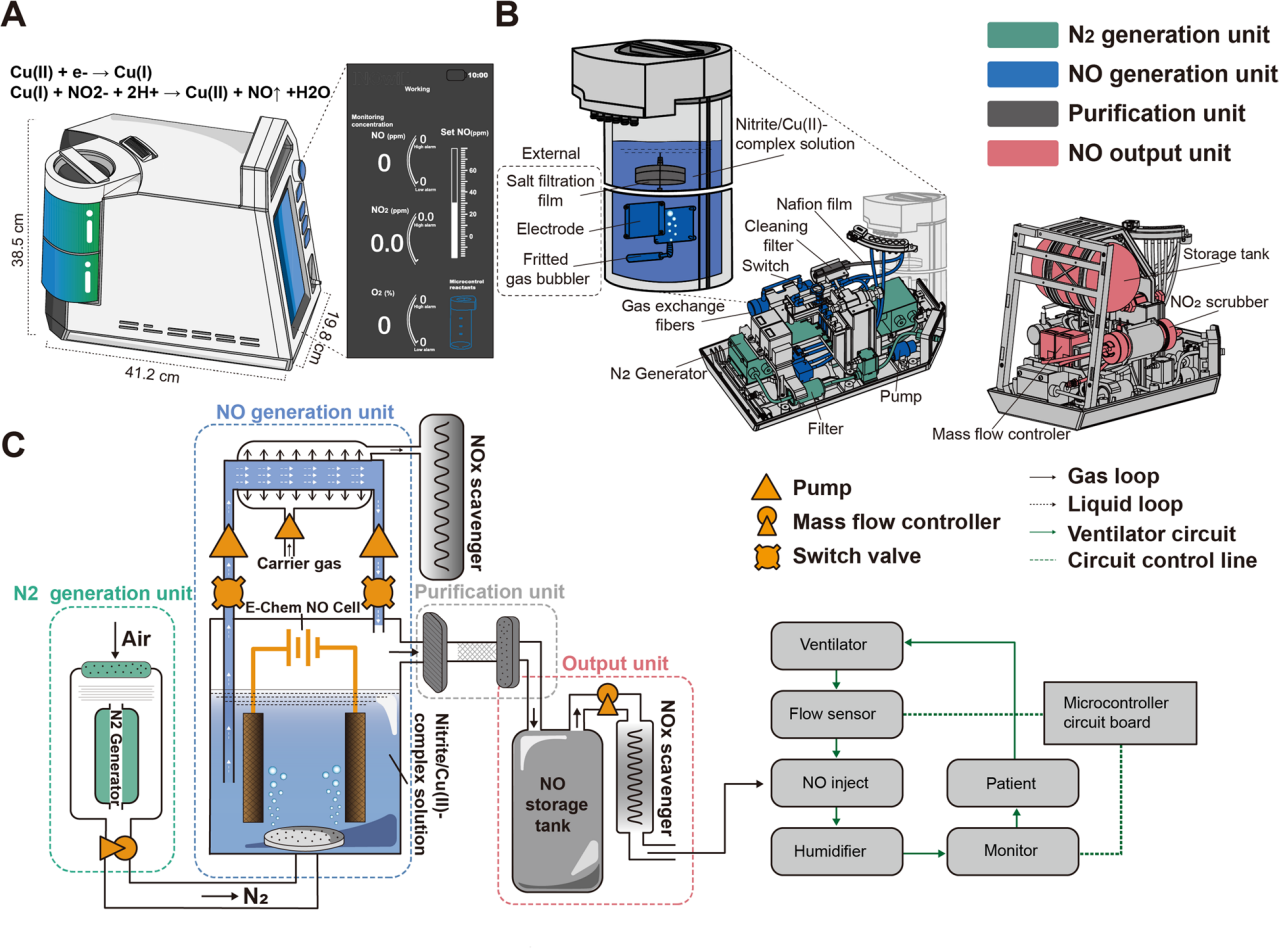
Design, configuration, and schematic of a novel ENG model. **A** Appearance, size, and configuration of the ENG. **B** Detailed positioning of each unit inside the highly integrated NO generator. **C** Schematic diagram of the ENG that consists of 4 sequentially connected units and two work modes controlled by a switch valve. The NO generator was connected to the ventilator pipeline and supervised by the circuit board

To meet the demands of NO in-home inhalation therapy in patients with PAH, we designed a controllable NO generator based on the electrochemical reduction of nitrite. In this novel device, substantial amounts of NO are electrochemically produced on the surface of an Au mesh electrode via reduction of a nitrite solution in the presence of copper (II)1,4,7-trimethyl-1,4,7-triazacyclononane (Cu(II)Me3TACN) without producing toxic byproducts. The ENG can rapidly produce high-purity NO with just one knob, which allows for bedside or in-home operation. To enable portable use, the NO generator had a very small design (41.2 cm × 38.5 cm × 19.8 cm) and only weighed 15 kg in total. This device is simple to operate with a single knob, and the real-time gas concentrations of NO, NO_2_, and O_2_ are displayed on a user-friendly interface (Fig. 1A).

The highly integrated ENG is composed of a nitrogen generation unit, a NO generation unit, a purification unit, and an output unit (Fig. 1B). The nitrogen generation unit is located at the bottom and contains a nitrogen-generating device and a filter device. The compressed air is filtered by a filter device to ensure that the dust, the particulate matter, or the aerosol has no influence on the ENG, and then the air enters the nitrogen-generating device, where oxygen (O_2_) and nitrogen (N_2_) are separated by their different permeation rates through the hollow fiber membrane. The NO generation unit is located next to the nitrogen generation unit and contains an external electrolytic module with a circular connection and a built-in gas exchange fiber. The electrolytic module is a consumable part that is easy to replace and has the capacity to generate NO at a volume equivalent to that of five 8 L NO cylinders. The gas exchange fiber is responsible for removing residual NO from the electrolyte. A purification unit consisting of a salt filtration film, Nafion film, and cleaning filter is located between the NO generation and output units to eliminate salt mist, moisture, and any impurities that may harm patients. The output unit is located at the top and includes a NO storage tank and a NO_2_ scrubber coated with ascorbic acid for reducing NO_2_ to NO.

The ENG can work in two modes (Fig. 1C). The first mode is for the rapid production of high-flow NO generated with a fritted gas bubbler. In this mode, the air is compressed and filtered into the nitrogen generation unit to achieve ≥99.0% N_2_, which is sprayed into the electrolytic cell through a purge to sweep away the NO generated in the electrolytic tank. The purified NO is mixed with N_2_ at a certain proportion in the storage tank, and the residual NO_2_ is reduced to NO in the NO_2_ scrubber. Then, NO is readily available for medical use. The ENG can be connected to a mask for direct inhalation or connected to the ventilator outlet that is supervised and regulated by a microcontroller. In the second mode, the electrolyte solution is directed from the electrolytic cell and flows through a reflux tube lined with a silicon fiber membrane, during which residual NO is extracted into the NOx absorbent. This extraction process is initiated by switching the valve and prevents chemical erosion of the electrode by residual NO in the solution when NO release is terminated.

Overall, our novel NO generator model was designed to synthesize, purify, recycle, and release NO in a compact and portable device. Given its small size and convenient replacement of consumables, this device has great potential for use in diverse clinical settings, including in-hospital treatment and in-home usage for patients with PAH.

### NO production efficiency and byproducts monitoring

**Fig. 2:**
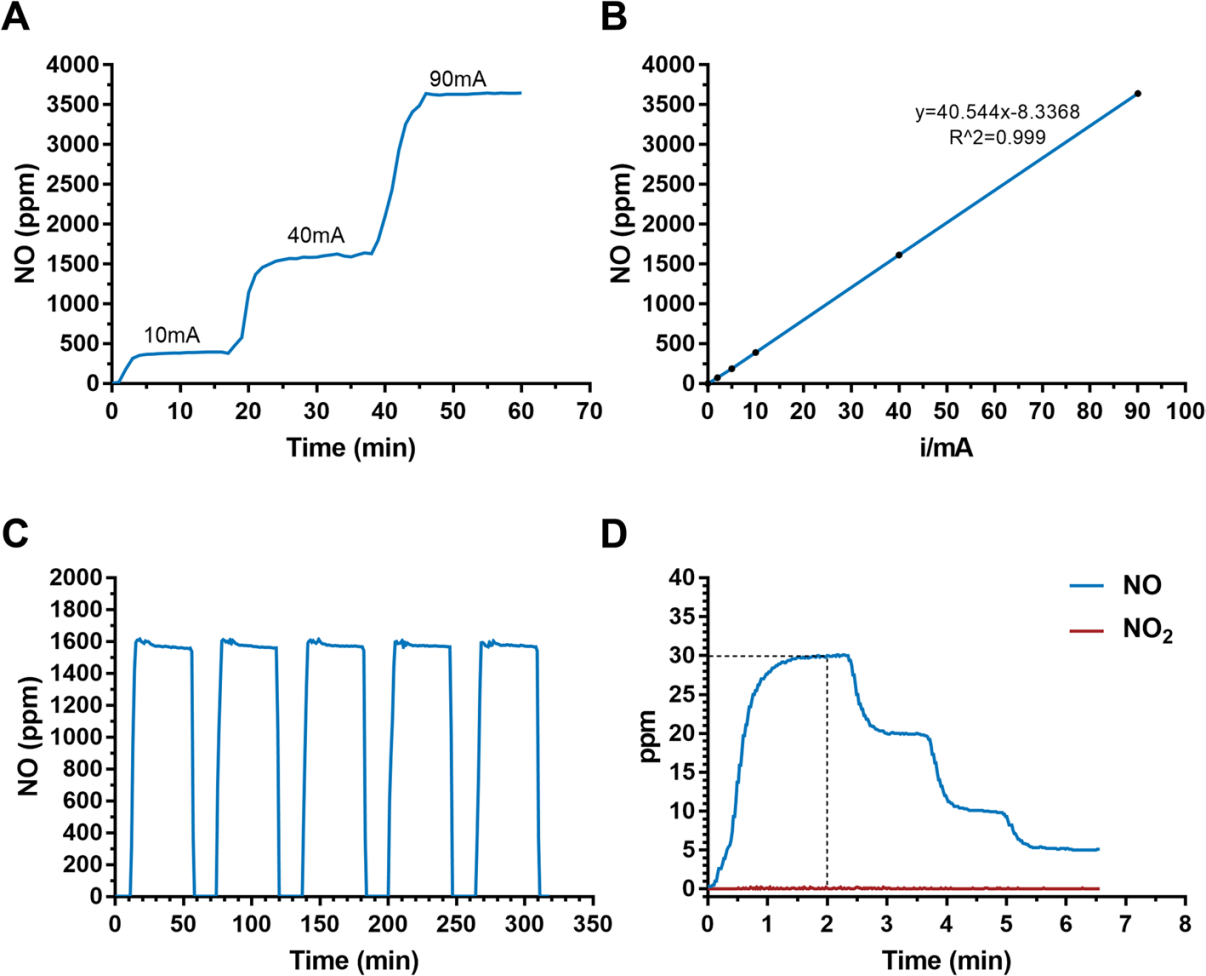
NO production and NO_2_ monitoring with the ENG. **A** NO concentration monitoring in the solution with different levels of applied current. **B** The linear relationship between the calibrated NO concentration and applied currents. **C** NO levels generated for five consecutive time periods. **D** NO_2_ levels under therapeutic doses of NO

We next tested the efficiency of NO production by applying consecutive currents from 10 to 90 mA on the electrodes in the ENG. A wide range of NO concentrations from 390 ppm to 3500 ppm was produced, and the switch time of NO production was less than 5 min (Fig. 2A). Importantly, the level of NO increased in a linear manner with increasing current applied to the electrodes, providing a theoretical basis for the sustained and precise release of NO (Fig. 2B). To understand the actual gas production efficiency, we tested the Faraday efficiency. With 40 mA current, the Faraday efficiency is about 74%. The value decreases with higher current and varies between 61~74%. Moreover, the electrolytic cell could generate a constant level of NO for five consecutive time periods (Fig. 2C), indicating its excellent reliability for the precise control of NO production at a fixed concentration. To confirm the generation rate and safety of the released NO, we monitored NO and NO_2_ simultaneously at the terminus of the pipeline. The results showed that ENG could reach the desired dose of NO within 2 min and NO_2_ was at an extremely low concentration (< 1 ppm) regardless of the therapeutic NO dose (Fig. 2D). Moreover, NO_2_ also remained at extremely low levels under a wider range of generated NO (Additional file 1: Table S1, Figure S1). According to ISO 18562:2017 biocompatibility evaluation of breathing gas pathways in healthcare applications, we further tested the emission of VOCs and particles at the terminus of the pipeline. The results showed that VOCs were below the threshold of toxicological concerns for the patient with different ages (Additional file 1: Table S2-S5). Moreover, it was shown that the particles were less than 10μg/m^3^, which is also in the safety ranges (Additional file 1: Table S6).

Taken together, these results demonstrated that our ENG has an excellent capacity for efficient, sustainable, and stable NO production, with a wide adjustment range and minimal byproducts production.

### Establishment of a porcine model of PAH

**Fig. 3:**
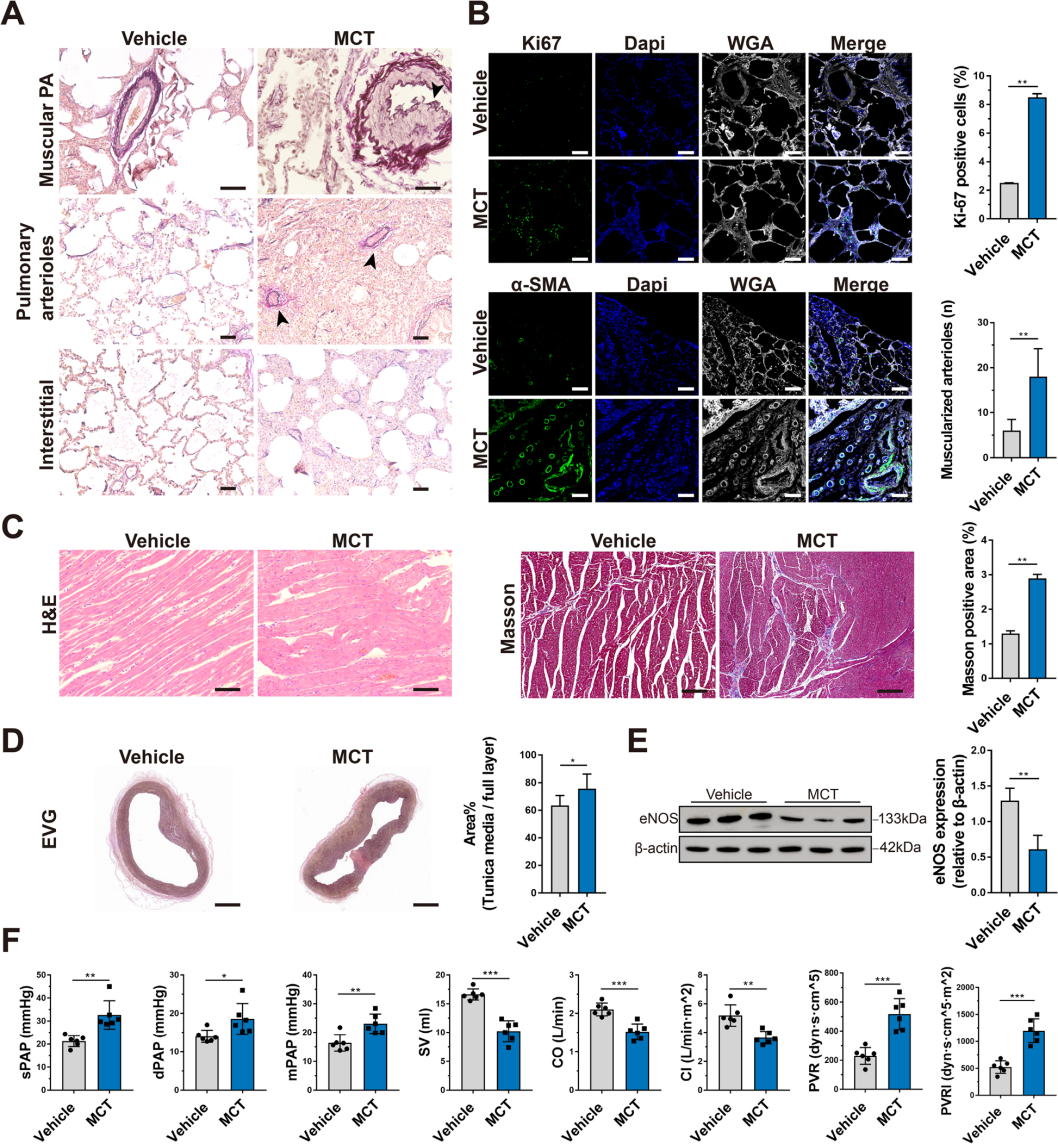
Establishment of a porcine PAH model. **A** EVG elastic staining of the lungs shows the pathological changes in piglets with MCT-induced PAH and the vehicle-treated group. The arrows indicate uneven thickening of the middle layer of the pulmonary arteries, with lumen stenosis, muscularized pulmonary arterioles, and lung interstitial thickening with inflammatory cell infiltration. **B** Immunofluorescence staining of Ki67 and α-SMA in paraffin-embedded sections of lung tissue from piglets with MCT-induced PAH or the vehicle-treated group. Scale bars, 50 μm. **C** H&E staining and Masson staining of the right ventricular myocardium of piglets with MCT-induced PAH or the vehicle-treated group. Scale bars, 50 μm for H&E staining and 100 μm for Masson staining. **D** EVG staining of the tunica media of the main pulmonary artery of piglets with MCT-induced PAH or the vehicle-treated group. Scale bars, 2 mm. **E** Representative images and quantification of eNOS expression in the lung tissues of piglets with MCT-induced PAH or the vehicle-treated group by western blotting. **F** Hemodynamic data for the pulmonary circulation, including sPAP, dPAP, mPAP, SV, CO, CI, PVR, and PVRI, were measured and calculated by catheter intervention in piglets with MCT-induced PAH or the vehicle-treated group. * *P*<0.05, ** *P*<0.01, *** *P*<0.001; compared to the vehicle group, as analyzed by Student’s *t* test

To examine the efficacy of the ENG for treating PAH, we established a porcine model by intraperitoneal injection of 12 mg/kg MCT twice (Additional file 1: Table S7 and Table S8). EVG elastic staining of lung tissues in the MCT group revealed uneven thickening of the middle layer of the pulmonary arteries, accompanied by lumen stenosis (black arrow), much more muscularized small pulmonary arterioles (black arrows), and pulmonary interstitial hyperplasia with inflammatory cell infiltration (Fig. 3A). Ki67 (*P*<0.01, *n*=3) and α-SMA (*P*<0.01, *n*=3) immunofluorescence staining of lung tissues revealed significantly increased vascular wall proliferation and thickening of the pulmonary arterioles in the MCT group compared with the vehicle group (Fig. 3B). H&E staining of the right ventricle showed distinct edema and disorder of cardiomyocytes in the MCT group, and Masson staining revealed a higher burden of myocardial fibrosis in the MCT group (*P*<0.01, *n*=3) (Fig. 3C). Moreover, the MCT group also showed an extensively thickened tunica media in the main pulmonary artery (*P*<0.05, *n*=3) (Fig. 3D). In addition, western blotting showed that endothelial nitric oxide synthase (eNOS) levels were significantly decreased in the MCT group compared with the vehicle group, indicating that endothelial synthesis of endogenous NO was attenuated in PAH (*P*<0.01, *n*=3) (Fig. 3E). From a hemodynamic perspective, higher sPAP (*P*=0.002, *F*=1.637, df=10, *n*=6), dPAP (*P*=0.027, *F*=2.384, df=10, *n*=6), mPAP (*P*=0.004, *F*=0.509, df=10, *n*=6), PVR (*P*=0.001, *F*=3.66, df=10, *n*=6), and PVRI (*P*=0.001, *F*=2.656, df=10, *n*=6) and lower stroke volume (SV) (*P*=0.001, *F*=2.932, df=10, *n*=6), CO (*P*=0.001, *F*=0.097, df=10, *n*=6), and CI (*P*=0.001, *F*=6.585, df=10, *n*=6) were observed in the MCT group than in the vehicle group (Fig. 3F). These results suggest that the MCT-induced PAH porcine model was successfully established and could be used to evaluate the therapeutic effect of the ENG.

### The electrochemical NO generator could alleviate PAH in a porcine model

**Fig. 4:**
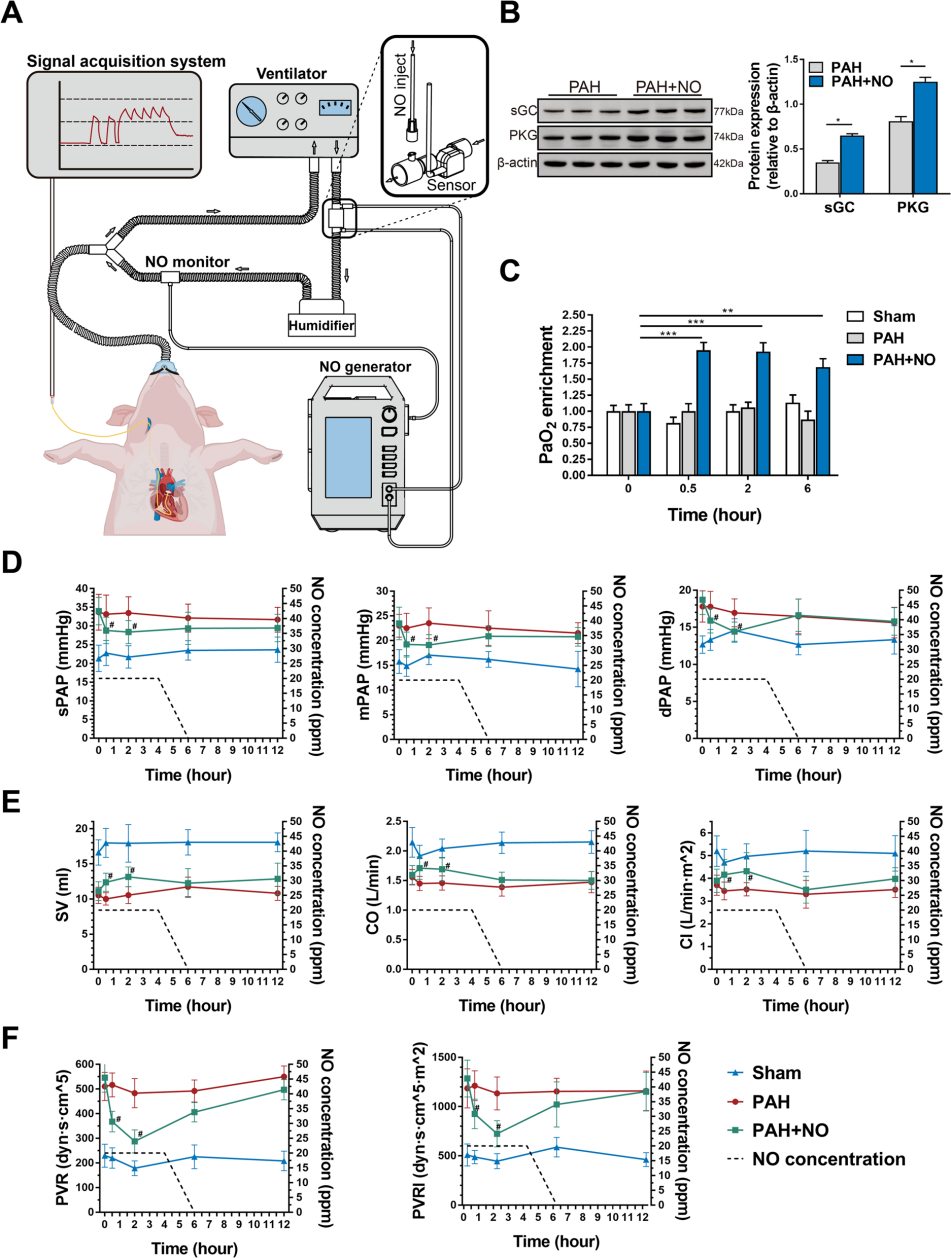
The ENG effectively alleviated hypoxemia and improved pulmonary hemodynamics in the porcine PAH model. **A** A schematic of the connection of the ENG to the ventilator and the acquisition of hemodynamic data. **B** Western blotting for sGC and PKG in the lung tissues of piglets with PAH in the presence or absence of NO inhalation (*n*=6/group). **C** PaO2 detection in piglets with PAH in the presence or absence of NO inhalation or the sham group at the indicated timepoint (*n*=6/group). **D**–**F** The measurement of PAP (**D**); SV, CO, and CI (**E**); and PVR and PVRI (**F**). * *P*<0.05, ** *P*<0.01; compared to the PAH group, as analyzed by Student’s *t* test (**B**) or two-way ANOVA with repeated measures (**C**–**F**)

By using a porcine model of PAH, we further explored whether the ENG could reduce PAP. We connected the ENG to a ventilator and recorded changes in PAP with a Swan-Ganz catheter before and after NO inhalation (Fig. 4A). In the PAH+NO group, animals inhaled NO at 20 ppm for 4 h as the initial dose, and the NO concentration decreased at a rate of 10 ppm per hour in the following 2 h. After NO inhalation, the expression of soluble guanylate cyclase (sGC) (*P*<0.05, *n*=3) and PKG (*P*<0.05, *n*=3) was significantly increased, indicating that inhaled NO activated the cGMP-mediated signaling pathway (Fig. 4B). As expected, arterial blood gas analysis showed that PaO2 enrichment was significantly increased compared to 0.5h (*P*<0.001), 2h (*P*<0.001), and 6h (*P*<0.01) after NO inhalation (Fig. 4C and Additional file 1: Table S9). Compared to the PAH group, the PAH+NO group showed significantly lower levels of sPAP (*P*=0.045 at 0.5h, *P*=0.032 at 2h), dPAP (*P*=0.05 at 0.5h, *P*=0.030 at 2h) and mPAP (*P*=0.036 at 0.5h, *P*=0.006 at 2h) at post-inhalation (Fig. 4D), accompanied by consistent improvements in SV (*P*=0.007 at 0.5h, *P*=0.003 at 2h), CO (*P*=0.005 at 0.5h, *P*=0.013 at 2h) and CI (*P*=0.026 at 0.5h, *P*=0.014 at 2h) (Fig. 4E). In addition, the calculated PVR (*P*=0.001 at 0.5h, *P*=0.001 at 2h) and PVRI (*P*=0.007 at 0.5h, *P*=0.001 at 2h) were alleviated at the same time point in the PAH+NO group (Fig. 4F).

**Fig. 5:**
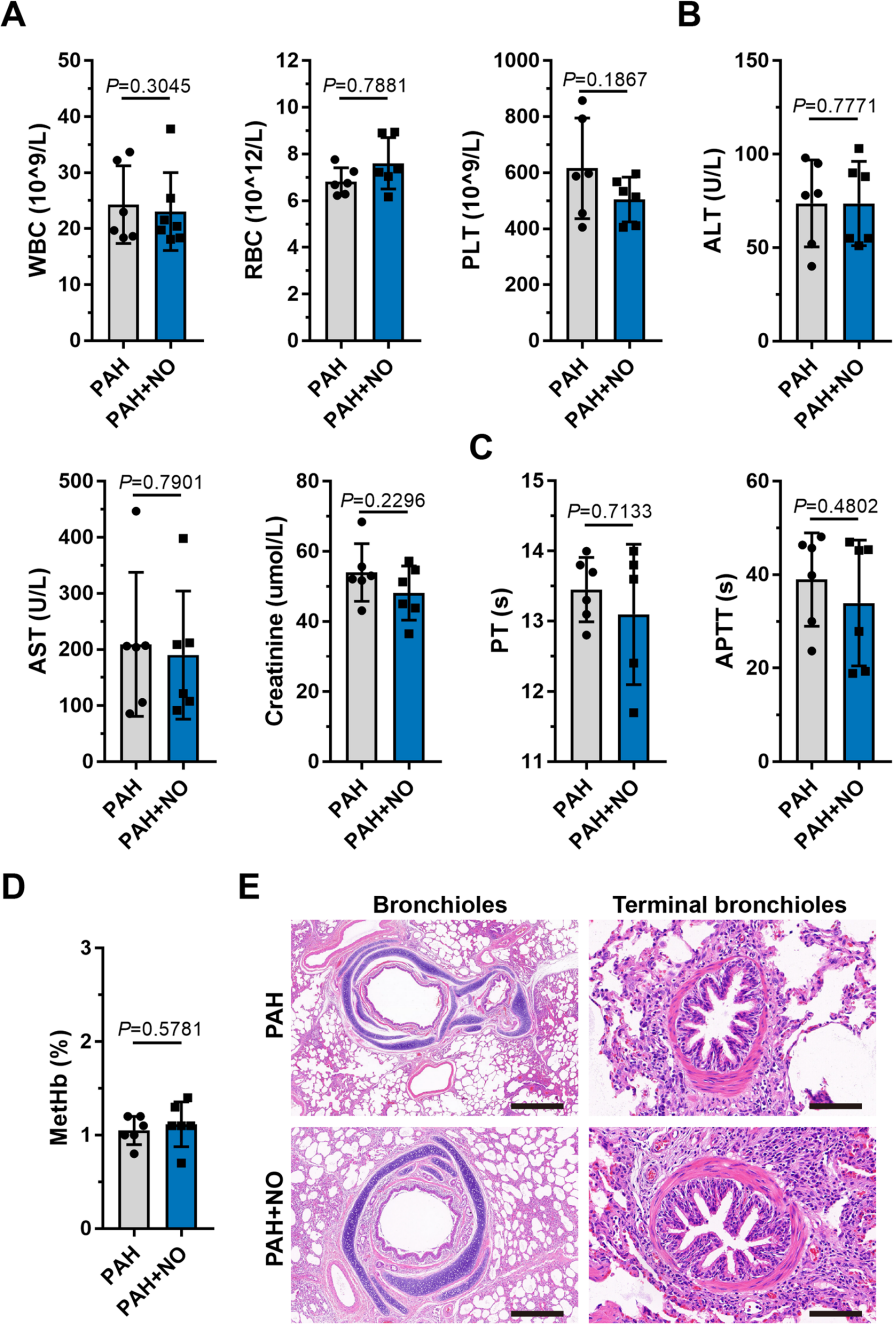
Examination of physiological indicators and lung pathology after NO inhalation with the ENG. **A**–**D** Comparison of blood cells (**A**); hepatic and renal function (**B**); procoagulant function (**C**); and methemoglobin (**D**) between piglets with PAH treated with the ENG and those not treated with the ENG; compared to the PAH group, as analyzed by Student’s *t* test. **E** H&E staining of paraffin sections of bronchioles (scale bars, 500 μm) and terminal bronchi (scale bars, 50 μm)

Blood tests, serum biochemistry, and coagulation examinations revealed no significant differences (*P*>0.05) before and after the application of the ENG (Fig. 5A–C, Additional file 1: Table S10), suggesting minimal hepatotoxicity and hematologic toxicity. After ENG treatment, methemoglobin in the PAH+NO group remained at a normal level and did not significantly increase when compared with that in the PAH group (*P*>0.05) (Fig. 5D), indicating that the therapeutic concentration of NO produced by the ENG did not cause methemoglobinemia. In pathology, the ENG did not cause significant injuries to lung bronchioles and terminal bronchi in PAH piglets (Fig. 5E).

Collectively, we confirmed in a porcine PAH model that the ENG can effectively alleviate hemodynamic compromise and improve cardiac functions at a therapeutic level of delivered NO without causing hematologic, hepatic disruptions, and lung injuries.

## Discussion

Currently, there are multiple designs of NO generator for inhalation therapy, but none are available for in-home use in the treatment of PAH [14]. Here, we designed a novel model of an NO generator that derives gaseous NO from a nitrite solution via an electrochemical method and verified its efficacy and safety in a preclinical model. The ENG delivered NO in a therapeutic range of 0–100 ppm in 5 min with negligible NO_2_ levels (<1 ppm), meeting the requirements of quality and safety set by the National Institute for Occupational Safety and Health [23]. The key electrolytic module in the ENG could maintain a steady output of NO at 20 ppm for at least 7 days, and the module could be easily replaced at low cost. In piglets with MCT-induced PAH, our device effectively improved hemodynamics and cardiac functions without causing major physiological disturbances.

Current medications used for the treatment of PAH, whether administered intravenously, subcutaneously, or orally, are associated dose-dependent systemic hypotension to varying extents. Of note, this adverse effect is absent with inhaled medications, including iloprost and NO. Iloprost is generally administered every 2-8 h with intermittent therapeutic effects, whereas NO can be inhaled for a relatively long time and maintains stable therapeutic effects. For in-home use of NO inhalation therapy, continuous low-dose inhalation during sleep-time could be a feasible option without interfering with patients at rest. To meet this goal, a reliable NO generator designed for in-home application is urgently needed.

Our device incorporated several important technical innovations to achieve this goal. First, the ENG uses N_2_ as the sweeping gas to eliminate the reaction between O_2_ and the newly formed NO, as well as to prevent the copper(II)-ligand catalyst from being oxidized in the solution near the electrodes. As a result, the efficiency of NO generation increased with a higher N_2_ sweeping flow. In the storage tank, the generated NO and N_2_ are pressurized to minimize the disproportionation of NO, and also enabled a stable and adjustable output of highly purified NO under different ventilator flow rates. We currently adjust the flow rate between 0.3 and 0.5 L/min, which is sufficient to meet the demands of most clinical settings. Second, a combination of an inert electrode (Au) and Cu-containing electrolyte solution was used for NO generation. When applying current at 0–90 mA, this method enabled the production of NO at concentrations ranging from 0 to 3600 ppm in the solution phase without producing toxic byproducts such as N_2_O, which greatly enhanced the safety of clinical application. Third, a silicone hollow fiber membrane was integrated into the gas exchange device, which effectively removes residual NO from the electrolyte solution. This modification improved the stability of the consumable part and prolonged its lifetime to achieve the desired NO level, significantly reducing the financial cost of the ENG.

Conventional high-pressure NO gas cylinders are limited by their high cost, bulkiness, and disproportionation of NO after storage over extended periods of time. To overcome these issues, many alternative solutions have been proposed, including miniature NO cylinders such as INOpulse, electric NO production with spark discharge, and the derivation of NO from chemicals such as liquid N_2_O_4_ [17, 18]. However, in-home application of miniature NO cylinders has a low acceptance rate in clinical settings due to its cumbersome installation procedure and frequent replacement of gas cylinders because of limited NO storage capacity. The electric NO generation device has difficulties in the maintenance of stable NO production due to fluctuating air flows through the sparker, and this device has a relatively higher risk of delivering toxic products, such as metal brass particles or O_3_. These safety and performance concerns restrict the utility of these devices for in-home therapy. Chemical-based generation of NO from liquid N_2_O_4_ is accompanied by the production of nitrogen oxide and toxic particles that cause health problems and may therefore not be a suitable option for in-home therapy. In this study, the NO gas released from our ENG model contained very few toxic byproducts, and the administration of NO using the ENG did not cause methemoglobinemia and had minimal hepatotoxicity and hematologic toxicity in piglets with experimental PAH. In this regard, our novel ENG device stands out due to its capacity to safely generate NO with minimal toxicity and a low cost of consumables, rendering it suitable for home management of PAH patients.

Due to the lack of sustainable and economic NO delivery devices at home, NO inhalation therapy has been primarily restricted to hospital settings and is not considered an in-home treatment option for PAH and other clinical scenarios. Our versatile, portable, and economical ENG model is not only suitable for bedside therapy for PAH (Additional file 1: Figure S2) but also has the potential to be used for patients with chronic lung diseases, congenital heart diseases, COVID-19 and even under some emergency circumstances. For example, the ENG could be deployed to rescue highland travelers with acute pulmonary edema due to its small size and rapid production of the desired NO concentration.

Several limitations of the ENG should be addressed before its successful translation to in-home medical use. First, the lack of an assembled battery may limit the extensive application of the ENG in emergency situations. Second, although the apparatus is smaller and weighs less than other products, it is not mobile enough for patients to receive the desired NO inhalation at any time, especially for highland travelers.

## Conclusions

A novel model of an NO generator was designed and tested, and it has the potential to generate high-purity NO for stable, continuous, and safe inhalational NO therapy for patients with PAH. The device is easy to use and can be operated by individuals without specialized training, which could greatly improve its acceptance rate in real-world practice. Our device may extend the indications for in-home inhalational NO therapy to other clinical scenarios, such as the treatment of COPD, high-altitude pulmonary edema, and COVID-19. Considering its high cost-effectiveness, our device has the possibility to address the unmet medical needs for a vast patient population in resource-limited countries around the world. Clinical trials are needed to test the clinical utility of this novel NO generator device.

## Supplementary Information

**Additional file 1: Table S1.** Gas composition produced from ENG. **Table S2.** Based on the daily respiratory volume of adults, calculated amount of discharged VOC that may be inhaled within different 24 hours. **Table S3.** Based on the daily respiratory volume of children, calculated amount of discharged VOC that may be inhaled within different 24 hours. **Table S4.** Based on the daily respiratory volume of infants, calculated amount of discharged VOC that may be inhaled within different 24 hours.**Table S5.** Based on the daily respiratory volume of the newborn, calculated amount of discharged VOC that may be inhaled within different 24 hours.**Table S6.** Total amount of particulate matter emission from the ENG gas outlet in 24 hours. **Table S7.** Preoperative blood routine, blood biochemistry and coagulation in vehicle group and MCT group. **Table S8.** Preoperative arterial blood gas analysis in vehicle group and MCT group.**Table S9.** Arterial blood gas analysis at 6 hours after NO inhalation in PAH group and PAH+NO group. **Table S10.** Postoperative blood routine, blood biochemistry and coagulation in PAH group and PAH+NO group. **Figure S1.** NO_2_ levels under a wider range of doses of NO (20-80ppm). **Figure S2.** Real-world picture of the ENG working with continuous airway positive pressure (CPAP) mask for bedside inhalation.

## Data Availability

The data that support the findings of this study are available from the corresponding author upon reasonable request.

## Abbreviations

PAH: Pulmonary artery hypertension
PAP: Pulmonary arterial pressure
cGMP: Cyclic guanosine monophosphate
PKG: Protein kinase G
eNOS: Endothelial nitric oxide synthase
sGC: Soluble guanylate cyclase
FDA: Food and Drug Administration
PPHN: Persistent pulmonary hypertension in neonates
COPD: Chronic obstructive pulmonary disease
ENG: Electrochemical NO Generator
VOCs: Volatile organic compounds
ISO: International standard organization
MCT: Monocrotaline
ppm: Parts per million
PVR: Pulmonary vascular resistance
PVRI: Pulmonary vascular resistance index
CO: Cardiac output
CI: Cardiac output index
SV: Stroke volume

## References

[1] Abman SH, Hansmann G, Archer SL, Ivy DD, Adatia I, Chung WK (2015). Pediatric pulmonary hypertension: guidelines from the American Heart Association and American Thoracic Society. Circulation..

[2] Barst RJ, McGoon MD, Elliott CG, Foreman AJ, Miller DP, Ivy DD (2012). Survival in childhood pulmonary arterial hypertension: insights from the registry to evaluate early and long-term pulmonary arterial hypertension disease management. Circulation..

[3] Walsh-Sukys MC, Tyson JE, Wright LL, Bauer CR, Korones SB, Stevenson DK (2000). Persistent pulmonary hypertension of the newborn in the era before nitric oxide: practice variation and outcomes. Pediatrics..

[4] Ignarro LJ, Ross G, Tillisch J (1991). Pharmacology of endothelium-derived nitric oxide and nitrovasodilators. West J Med.

[5] Frostell CG, Blomqvist H, Hedenstierna G, Lundberg J, Zapol WM (1993). Inhaled nitric oxide selectively reverses human hypoxic pulmonary vasoconstriction without causing systemic vasodilation. Anesthesiology..

[6] Clark RH, Kueser TJ, Walker MW, Southgate WM, Huckaby JL, Perez JA (2000). Low-dose nitric oxide therapy for persistent pulmonary hypertension of the newborn. N Engl J Med.

[7] Fox-Robichaud A, Payne D, Hasan SU, Ostrovsky L, Fairhead T, Reinhardt P (1998). Inhaled NO as a viable antiadhesive therapy for ischemia/reperfusion injury of distal microvascular beds. J Clin Invest.

[8] Date H, Triantafillou AN, Trulock EP, Pohl MS, Cooper JD, Patterson GA (1996). Inhaled nitric oxide reduces human lung allograft dysfunction. J Thorac Cardiovasc Surg.

[9] Vonbank K, Ziesche R, Higenbottam TW, Stiebellehner L, Petkov V, Schenk P (2003). Controlled prospective randomised trial on the effects on pulmonary haemodynamics of the ambulatory long term use of nitric oxide and oxygen in patients with severe COPD. Thorax..

[10] Kavarana MN, Pessin-Minsley MS, Urtecho J, Catanese KA, Flannery M, Oz MC (2002). Right ventricular dysfunction and organ failure in left ventricular assist device recipients: a continuing problem. Ann Thorac Surg.

[11] Kato GJ, Steinberg MH, Gladwin MT (2017). Intravascular hemolysis and the pathophysiology of sickle cell disease. J Clin Invest.

[12] Jr Roberts JD, Lang P, Bigatello LM, Vlahakes GJ, Zapol WM (1993). Inhaled nitric oxide in congenital heart disease. Circulation..

[13] Lisi F, Zelikin AN, Chandrawati R (2021). Nitric oxide to fight viral infections. Adv Sci (Weinh).

[14] Todd Tzanetos DR, Housley JJ, Barr FE, May WL, Landers CD (2015). Implementation of an inhaled nitric oxide protocol decreases direct cost associated with its use. Respir Care.

[15] Gianni S, Carroll RW, Kacmarek RM, Berra L (2021). Inhaled nitric oxide delivery systems for mechanically ventilated and nonintubated patients: a review. Respir Care.

[16] Nathan SD, Flaherty KR, Glassberg MK, Raghu G, Swigris J, Alvarez R (2020). Inhaled nitric oxide in subjects at risk of pulmonary hypertension associated with pulmonary fibrosis. Chest..

[17] Yu B, Muenster S, Blaesi AH, Bloch DB, Zapol WM (2015). Producing nitric oxide by pulsed electrical discharge in air for portable inhalation therapy. Sci Transl Med.

[18] Center for Drug Evaluation and Research. Application number: 202860Orig1s000. https://www.accessdata.fda.gov/drugsatfda_docs/nda/2019/202860Orig1s000Approv.pdf. Accessed 20 Sep 2022.

[19] The ARRIVE guidelines 2.0: author checklist. https://arriveguidelines.org/resources/author-checklists. Accessed 4 Nov 2022.

[20] Biocompatibility evaluation of breathing gas pathways in healthcare applications — Part 3: Tests for emissions of volatile organic compounds (VOCs) (ISO 18562-3:2017). https://www.iso.org/standard/62894.html. Accessed 20 Sep 2022.

[21] Biocompatibility evaluation of breathing gas pathways in healthcare applications — Part 1: Evaluation and testing within a risk management process (ISO 18562-1:2017). https://www.iso.org/standard/62892.html. Accessed 20 Sep 2022.

[22] Biocompatibility evaluation of breathing gas pathways in healthcare applications — Part 2: Tests for emissions of particulate matter (ISO 18562-2:2017). https://www.iso.org/standard/62893.html. Accessed 20 Sep 2022.

[23] The National Institute for Occupational Safety and Health (NIOSH). NITROGEN DIOXIDE. https://www.cdc.gov/niosh/pel88/10102-44.html. Accessed 20 Sept 2022.

