# Supplementary material for: An electrochemical nitric oxide generator for in-home inhalation therapy in pulmonary artery hypertension
Source: BMC Med. 2022 Dec 15;20:481. doi: 10.1186/s12916-022-02686-6 (PMC9753075; doi:10.1186/s12916-022-02686-6)
Supplement: Supplementary file 1 — Additional file 1: Table S1. Gas composition produced from ENG. Table S2. Based on the daily respiratory volume of adults, calculated amount of discharged VOC that may be inhaled within different 24 hours. Table S3. Based on the daily respiratory volume of children, calculated amount of discharged VOC that may be inhaled within different 24 hours. Table S4. Based on the daily respiratory volume of infants, calculated amount of discharged VOC that may be inhaled within different 24 hours.Table S5. Based on the daily respiratory volume of the newborn, calculated amount of discharged VOC that may be inhaled within different 24 hours.Table S6. Total amount of particulate matter emission from the ENG gas outlet in 24 hours. Table S7. Preoperative blood routine, blood biochemistry and coagulation in vehicle group and MCT group. Table S8. Preoperative arterial blood gas analysis in vehicle group and MCT group.Table S9. Arterial blood gas analysis at 6 hours after NO inhalation in PAH group and PAH+NO group. Table S10. Postoperative blood routine, blood biochemistry and coagulation in PAH group and PAH+NO group. Figure S1. NO2 levels under a wider range of doses of NO (20-80ppm). Figure S2. Real-world picture of the ENG working with continuous airway positive pressure (CPAP) mask for bedside inhalation. [file 12916_2022_2686_MOESM1_ESM.docx]

**Additional File 1**

**An Electrochemical Nitric Oxide Generator for In-home Inhalation Therapy in Pulmonary Artery Hypertension**

Yiwei Liu, Yifan Zhu, Chenyu Jiang, Zhanhao Su, Yi Yan, Bei Feng, Wen Mao, Yuyan Zhang, Xiaojian Wang, Zhuoming Xu and Hao Zhang.

**Table S1.** Gas composition produced from ENG.

**Table S2.** Based on the daily respiratory volume of adults, calculated amount of discharged VOC that may be inhaled within different 24 hours.

**Table S3.** Based on the daily respiratory volume of children, calculated amount of discharged VOC that may be inhaled within different 24 hours.

**Table S4.** Based on the daily respiratory volume of infants, calculated amount of discharged VOC that may be inhaled within different 24 hours.

**Table S5.** Based on the daily respiratory volume of the newborn, calculated amount of discharged VOC that may be inhaled within different 24 hours.

**Table S6.** Total amount of particulate matter emission from the ENG gas outlet in 24 hours.

**Table S7.** Preoperative blood routine, blood biochemistry and coagulation in vehicle group and MCT group.

**Table S8.** Preoperative arterial blood gas analysis in vehicle group and MCT group.

**Table S9.** Arterial blood gas analysis at 6 hours after NO inhalation in PAH group and PAH+NO group.

**Table S10.** Postoperative blood routine, blood biochemistry and coagulation in PAH group and PAH+NO group.

**Figure S1.** NO_2_ levels under a wider range of doses of NO (20-80ppm)

**Figure S2.** Real-world picture of the ENG working with continuous airway positive pressure (CPAP) mask for bedside inhalation.

**Table S1.** **Gas composition produced from ENG.**

| Test item | Unit | Detection limit | Results |
| --- | --- | --- | --- |
| N_2_ | % (v/v) | / | 99.91 |
| NO | 10-6 (v/v) | 5 | 1000 |
| NO_2_ | 10-6 (v/v) | 2.5 | ND |
| N_2_O | 10-6 (v/v) | 0.05 | ND |
| CO_2_ | 10-6 (v/v) | 0.05 | ND |
| O_2_ | 10-6 (v/v) | 0.01 | 4.30 |

N_2_, nitrogen; NO, nitric oxide; NO_2_, nitrogen dioxide; N_2_O, nitrous oxide; CO_2_, carbon dioxide; O_2_, oxygen; ND, not detected.

**Table S2.** **Based on the daily respiratory volume of adults, calculated amount of discharged VOC that may be inhaled within different 24 hours.**

| Compounds | Initial run | 24 hours | 168 hours |
| --- | --- | --- | --- |
| Benzene | ＜40μg/d | ＜40μg/d | ＜40μg/d |
| Methylbenzene | ＜40μg/d | ＜40μg/d | ＜40μg/d |
| Ethylbenzene | ＜40μg/d | ＜40μg/d | ＜40μg/d |
| Paraxylene | ＜40μg/d | ＜40μg/d | ＜40μg/d |
| Meta-Xylene | ＜40μg/d | ＜40μg/d | ＜40μg/d |
| O-xylene | ＜40μg/d | ＜40μg/d | ＜40μg/d |
| Styrene | ＜40μg/d | ＜40μg/d | ＜40μg/d |
| Butyl acetate | ＜40μg/d | ＜40μg/d | ＜40μg/d |
| N-undecane | ＜40μg/d | ＜40μg/d | ＜40μg/d |
| Every other VOCs | ＜40μg/d | ＜40μg/d | ＜40μg/d |

VOC, volatile organic compounds.

**Table S3. Based on the daily respiratory volume of children, calculated amount of discharged VOC that may be inhaled within different 24 hours.**

| Compounds | | Initial run | 24 hours | 168 hours |
| --- | --- | --- | --- | --- |
| Benzene | ＜10μg/d | | ＜10μg/d | ＜10μg/d |
| Methylbenzene | ＜10μg/d | | ＜10μg/d | ＜10μg/d |
| Ethylbenzene | ＜10μg/d | | ＜10μg/d | ＜10μg/d |
| Paraxylene | ＜10μg/d | | ＜10μg/d | ＜10μg/d |
| Meta-Xylene | ＜10μg/d | | ＜10μg/d | ＜10μg/d |
| O-xylene | ＜10μg/d | | ＜10μg/d | ＜10μg/d |
| Styrene | ＜10μg/d | | ＜10μg/d | ＜10μg/d |
| Butyl acetate | ＜10μg/d | | ＜10μg/d | ＜10μg/d |
| N-undecane | ＜10μg/d | | ＜10μg/d | ＜10μg/d |
| Every other VOCs | ＜10μg/d | | ＜10μg/d | ＜10μg/d |

VOC, volatile organic compounds.

**Table S4. Based on the daily respiratory volume of infants, calculated amount of discharged VOC that may be inhaled within different 24 hours.**

| Compounds | Initial run | 24 hours | 168 hours |
| --- | --- | --- | --- |
| Benzene | ＜4μg/d | ＜4μg/d | ＜4μg/d |
| Methylbenzene | ＜4μg/d | ＜4μg/d | ＜4μg/d |
| Ethylbenzene | ＜4μg/d | ＜4μg/d | ＜4μg/d |
| Paraxylene | ＜4μg/d | ＜4μg/d | ＜4μg/d |
| Meta-Xylene | ＜4μg/d | ＜4μg/d | ＜4μg/d |
| O-xylene | ＜4μg/d | ＜4μg/d | ＜4μg/d |
| Styrene | ＜4μg/d | ＜4μg/d | ＜4μg/d |
| Butyl acetate | ＜4μg/d | ＜4μg/d | ＜4μg/d |
| N-undecane | ＜4μg/d | ＜4μg/d | ＜4μg/d |
| Every other VOCs | ＜4μg/d | ＜4μg/d | ＜4μg/d |

VOC, volatile organic compounds.

**Table S5. Based on the daily respiratory volume of the newborn, calculated amount of discharged VOC that may be inhaled within different 24 hours.**

| Compounds | Initial run | 24 hours | 168 hours |
| --- | --- | --- | --- |
| Benzene | ＜0.42μg/d | ＜0.42μg/d | ＜0.42μg/d |
| Methylbenzene | ＜0.42μg/d | ＜0.42μg/d | ＜0.42μg/d |
| Ethylbenzene | ＜0.42μg/d | ＜0.42μg/d | ＜0.42μg/d |
| Paraxylene | ＜0.42μg/d | ＜0.42μg/d | ＜0.42μg/d |
| Meta-Xylene | ＜0.42μg/d | ＜0.42μg/d | ＜0.42μg/d |
| O-xylene | ＜0.42μg/d | ＜0.42μg/d | ＜0.42μg/d |
| Styrene | ＜0.42μg/d | ＜0.42μg/d | ＜0.42μg/d |
| Butyl acetate | ＜0.42μg/d | ＜0.42μg/d | ＜0.42μg/d |
| N-undecane | ＜0.42μg/d | ＜0.42μg/d | ＜0.42μg/d |
| Every other VOCs | ＜0.42μg/d | ＜0.42μg/d | ＜0.42μg/d |

VOC, volatile organic compounds.

**Table S6. Total amount of particulate matter emission from the ENG gas outlet in 24 hours.**

| Particle size range | Average concentration (μg/m^3^) | | | |
| --- | --- | --- | --- | --- |
|  | **First 8h** | **Second 8h** | **Third 8h** | **Total 24h** |
| ≤2.5 μm | ＜10 | ＜10 | ＜10 | ＜10 |
| ≤10 μm | ＜10 | ＜10 | ＜10 | ＜10 |

ENG, Electrochemical Nitric Oxide Generator.

**Table S7. Preoperative blood routine, blood biochemistry and coagulation in vehicle group and MCT group.**

| Characteristics | Vehicle (n=6) | MCT (n=12) | *P* value |
| --- | --- | --- | --- |
| WBC (10^9/L) | 18.2±1.3 | 18.9±4.5 | 0.884 |
| RBC (10^12/L) | 6.2±1.0 | 7.4±0.3* | 0.026 |
| HCT (%) | 28.5±3.3 | 33.9±3.7 | 0.050 |
| MCH (pg) | 14.4±0.9 | 14.4±1.0 | 0.999 |
| MCHC (%) | 309.3±17.5 | 312.7±9.1 | 0.693 |
| MCV (fl) | 46.3±3 | 45.9±3.5 | 0.839 |
| HGB (g/L) | 88.8±14.8 | 105.8±10.8 | 0.067 |
| RET (%) | 2.0±2.5 | 1.1±1.0 | 0.432 |
| PLT (10^9/L) | 680.5±185.1 | 706.3±148.2 | 0.812 |
| LYM (%) | 55.6±9.1 | 42.11±21.7 | 0.281 |
| Neutrophil (%) | 24.0±7.4 | 29.9±9.2 | 0.327 |
| TP (g/L) | 66.3±3.4 | 64.0±4.5 | 0.425 |
| ALB (g/L) | 28.1±4.3 | 28.1±2.7 | 0.999 |
| ALB/GLB | 0.75±0.19 | 0.8±0.14 | 0.645 |
| ALT (U/L) | 37.3±6.9 | 43.3±7.1 | 0.217 |
| AST (U/L) | 32.3±4.0 | 54.0±14.4* | 0.027 |
| GGT (U/L) | 41.3±9.5 | 52.0±6.3 | 0.061 |
| UREA (mmol/L) | 2.1±0.5 | 1.9±0.6 | 0.663 |
| CRE (μmoI/L) | 57.4±15.1 | 52.45±6.9 | 0.493 |
| GLU (mmol/L) | 5.9±3.0 | 5.2±1.3 | 0.643 |
| PT (seconds) | 12.8±0.5 | 12.6±0.1 | 0.454 |
| APTT (seconds) | 22.0±3.2 | 32.7±9.5 | 0.073 |
| FIB (g/L) | 2.3±0.3 | 2.2±0.6 | 0.716 |

MCT, monocrotaline; WBC, white blood cell; RBC, red blood cell; HCT, hematocrit value; MCH, mean corpuscular hemoglobin; MCHC, mean corpuscular hemoglobin concentration; MCV, mean corpuscular volume; HGB, hemoglobin; RET, reticulocyte; PLT, platelet; LYM, lymphocyte; TP, total protein; ALB, albumin; GLB, globulin; ALT, alanine aminotransferase; AST, aspartate aminotransferase; GGT, gamma-glutamyl transferase; UREA, blood urea; CRE, creatinine; GLU, glucose; PT, prothrombin time; APTT, activated partial thromboplastin time; FIB, plasma fibrinogen; Data are mean ± SD; Statistical significance was determined using Student’s t-test (**P* <0.05).

**Table S8. Preoperative arterial blood gas analysis in vehicle group and MCT group.**

| Characteristics | Vehicle (n=6) | MCT (n=12) | P value |
| --- | --- | --- | --- |
| PH | 7.4±0.2 | 7.3±0.1 | 0.188 |
| PaO_2_ (mmHg) | 419.0±92.7 | 323.5±74.5* | 0.031 |
| PaCO_2_ (mmHg) | 41.1±15.1 | 69.5±24.5* | 0.026 |
| K^+^ (mmol/L) | 4.2±0.1 | 4.8±1.1 | 0.092 |
| Na^+^ (mmol/L) | 138.3±1.3 | 137.8±4.7 | 0.897 |
| Ca^2+^ (mmol/L) | 1.31±0.03 | 1.34±0.07 | 0.213 |
| Cl^-^ (mmol/L) | 105.0±3.5 | 100.5±7.1 | 0.247 |
| SBE (mmol/L) | 1.9±2.8 | 3.5±4.0 | 0.373 |
| SB (mmol/L) | 28.0±1.0 | 27.4±3.1 | 0.499 |

MCT, monocrotaline; SBE, standard base excess; SB, standard bicarbonate; Data are mean ± SD; Statistical significance was determined using Student’s t-test (**P* <0.05).

**Table S9. Arterial blood gas analysis at 6 hours after NO inhalation in PAH group and PAH+NO group.**

| **Characteristics** | **PAH (n=6)** | **PAH+NO (n=6)** | **P value** |
| --- | --- | --- | --- |
| PH | 7.4±0.3 | 7.4±0.2 | 0.955 |
| PaO_2_ (mmHg) | 319.3±117.7 | 461.7.8±74.0* | 0.031 |
| PaCO_2_ (mmHg) | 48.8±11.0 | 38.9±6.3 | 0.086 |
| K^+^ (mmol/L) | 5.1±1.7 | 5.1±1.4 | 0.971 |
| Na^+^ (mmol/L) | 131.8±2.7 | 132.2±6.8 | 0.910 |
| Ca^2+^ (mmol/L) | 1.29±0.02 | 1.30±0.07 | 0.743 |
| Cl^-^ (mmol/L) | 98.0±1.9 | 92.2±5.4 | 0.073 |
| SBE (mmol/L) | 4.8±1.3 | 10.7±1.9* | 0.000 |
| SB (mmol/L) | 28.9±1.1 | 33.3±1.7* | 0.003 |

PAH, pulmonary artery hypertension; NO, nitric oxide; SBE, standard base excess; SB, standard bicarbonate; Data are mean ± SD; Statistical significance was determined using Student’s t-test (**P* <0.05).

**Table S10. Postoperative blood routine, blood biochemistry and coagulation in PAH group and PAH+NO group.**

| **Characteristics** | **PAH (n=6)** | **PAH+NO (n=6)** | **P value** |
| --- | --- | --- | --- |
| WBC (10^9/L) | 24.3±6.9 | 23.1±6.9 | 0.305 |
| RBC (10^12/L) | 6.8±0.6 | 7.5±1.7 | 0.788 |
| HCT (%) | 31.2±3.1 | 35.6±8.1 | 0.225 |
| MCH (pg) | 14.3±0.9 | 14.7±1.0 | 0.445 |
| MCHC (%) | 313.2±1.5 | 310.7±12.5 | 0.669 |
| MCV (fl) | 45.7±2.9 | 47.5±4.2 | 0.396 |
| HGB (g/L) | 97.7±9.5 | 111.0±25.7 | 0.248 |
| RET (%) | 0.6±0.5 | 0.7±0.6 | 0.851 |
| PLT (10^9/L) | 616.0±180.1 | 497.1±64.7 | 0.187 |
| LYM (%) | 35.3±6.5 | 35.8±8.5 | 0.901 |
| Neutrophil (%) | 49.5±10.1 | 48.7±10.5 | 0.887 |
| TP (g/L) | 65.0±3.6 | 67.2±5.9 | 0.451 |
| ALB (g/L) | 28.9±2.9 | 31.3±5.2 | 0.322 |
| ALB/GLB | 0.8±0.19 | 0.9±0.3 | 0.343 |
| ALT (U/L) | 73.6±23.2 | 73.7±20.3 | 0.777 |
| AST (U/L) | 209.3±128.4 | 174.4±122.6 | 0.790 |
| GGT (U/L) | 48.2±7.5 | 48.8±9.1 | 0.894 |
| UREA (mmol/L) | 3.0±1.0 | 3.7±1.6 | 0.419 |
| CRE (μmoI/L) | 54.0±8.2 | 49.7±7.5 | 0.301 |
| GLU (mmol/L) | 5.5±1.5 | 5.4±0.9 | 0.887 |
| PT (seconds) | 13.5±0.5 | 13.1±0.7 | 0.713 |
| APTT (seconds) | 38.9±9.9 | 33.0±12.2 | 0.480 |
| FIB (g/L) | 3.8±0.3 | 3.7±0.6 | 0.611 |

PAH, pulmonary artery hypertension; NO, nitric oxide; WBC, white blood cell; RBC, red blood cell; HCT, hematocrit value; MCH, mean corpuscular hemoglobin; MCHC, mean corpuscular hemoglobin concentration; MCV, mean corpuscular volume; HGB, hemoglobin; RET, reticulocyte; PLT, platelet; LYM, lymphocyte; TP, total protein; ALB, albumin; GLB, globulin; ALT, alanine aminotransferase; AST, aspartate aminotransferase; GGT, gamma-glutamyl transferase; UREA, blood urea; CRE, creatinine; GLU, glucose; PT, prothrombin time; APTT, activated partial thromboplastin time; FIB, plasma fibrinogen; Data are mean ± SD; Statistical significance was determined using Student’s t-test (**P* <0.05).


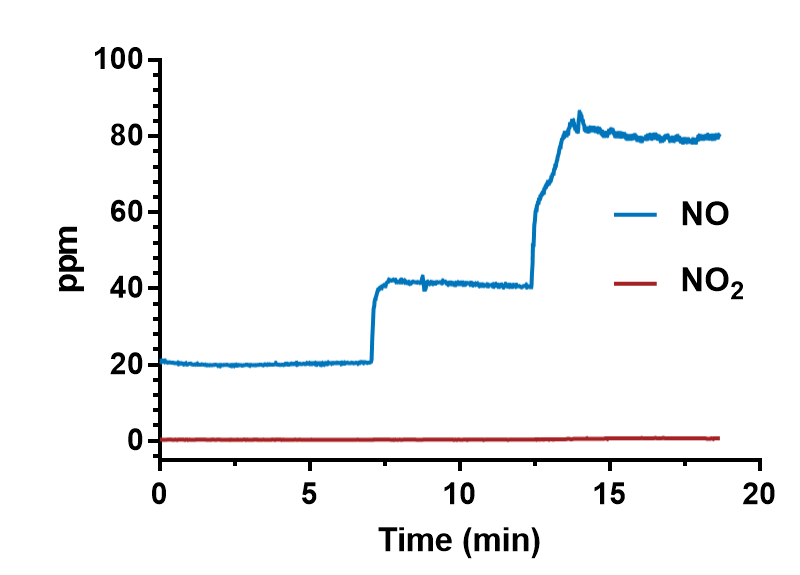


**Figure S1.** NO_2_ levels under high dose of NO (20-80ppm)


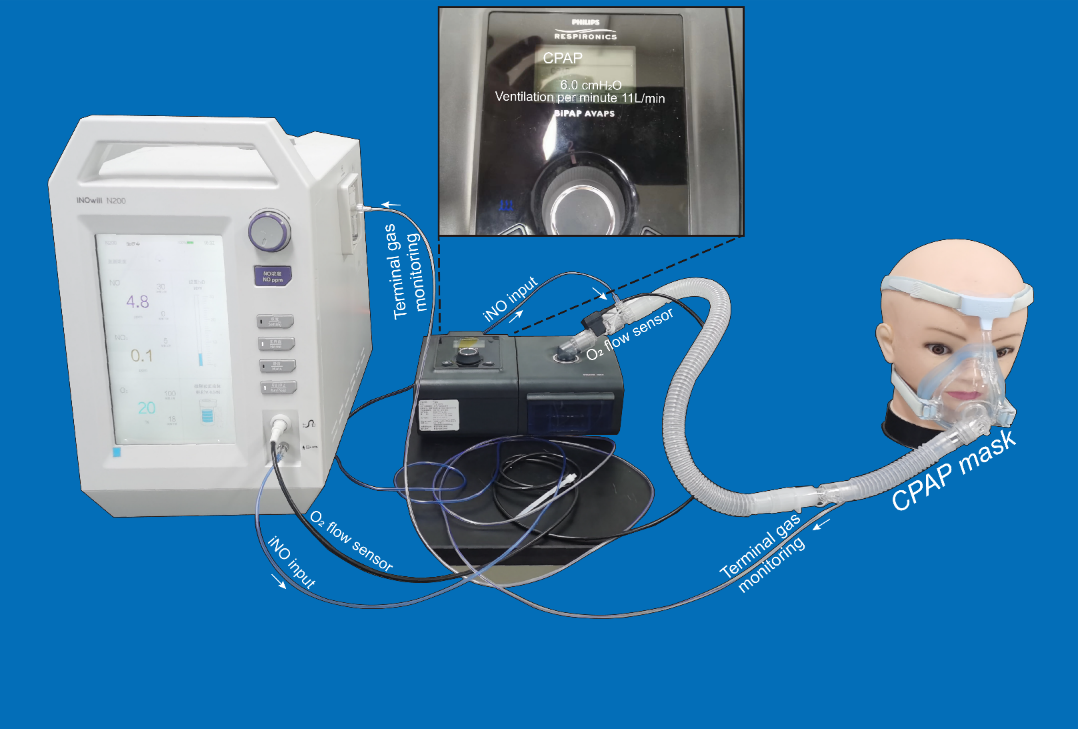


**Figure S2.** Real-world picture of the ENG working with continuous airway positive pressure (CPAP) mask for bedside NO inhalation therapy.
